# Comparison of Nursing Care Quality Between Team‐Based Primary Nursing and Case Method Approaches in Patients With Hematological Malignancies

**DOI:** 10.1155/jonm/9634361

**Published:** 2026-11-09

**Authors:** Shiva Salar, Raziyeh Ghafouri, Malihe Nasiri

**Affiliations:** ^1^ Department of Medical Surgical Nursing Student Research Committee School of Nursing & Midwifery Shahid Beheshti University of Medical Sciences Tehran Iran sbmu.ac.ir; ^2^ Department of Medical Surgical Nursing School of Nursing & Midwifery Shahid Beheshti University of Medical Sciences Tehran Iran sbmu.ac.ir; ^3^ Department of Basic Sciences School of Nursing & Midwifery Shahid Beheshti University of Medical Sciences Tehran Iran sbmu.ac.ir

**Keywords:** case method, hematological malignancies, primary nursing, quality of nursing care, quality of oncology nursing care

## Abstract

**Background:**

Cancer remains the leading cause of mortality worldwide. Patients with cancer face numerous challenges throughout their treatment, partly because their care must be the individualized. Because nursing care delivery models can affect the quality of care, this study aimed to compare the quality of nursing care provided through the team‐based primary nursing and the case method approaches in patients with hematological malignancies.

**Methods:**

This descriptive‐comparative study included patients over than 18 years who had been hospitalized for more than 48 h and were able to read. Data were collected through convenience sampling between October 2024 and March 2025 using the Quality of Oncology Nursing Care Scale (QONCS).

**Results:**

A total of 173 patients were enrolled in the study, comprising 86 individuals admitted to the hematology ward and 87 to the bone marrow transplantation (BMT) unit. The overall Quality of Oncology Nursing Care was significantly higher in the BMT unit (121.69 ± 16.97 vs. 110.53 ± 22.88; *p* = 0.003). Among the care dimensions, compassionate care and respect for patients’ beliefs were significantly higher in the BMT group, whereas spiritual care did not differ significantly between the groups.

**Conclusion:**

The mean quality of oncology nursing care was higher under the team‐based primary nursing model than under the case method. These findings suggest that team‐based primary nursing may be associated whit a more effective approach for improving the quality of care delivered to patients with hematological malignancies.

**Implication for Nursing Management:**

Patient‐reported nursing care quality may be associated with implementing team‐based primary nursing, particularly in patient‐centered dimensions. Nursing managers and policymakers should consider care delivery models that promote the continuity of care, clear responsibilities, and a comprehensive understanding of patients’ physical, psychosocial, and spiritual needs. Given the relatively low ratings for psychosocial and spiritual care, targeted strategies to strengthen these dimensions are warranted. However, differences in care quality should be interpreted cautiously, as factors beyond the nursing care model may also contribute to the observed findings.

## 1. Introduction

Cancer is the second leading cause of mortality worldwide [1], posing a serious threat to human health and life expectancy [2]. It is a rapidly growing global concern, evidenced by over 19 million new cases and more than 10 million deaths in 2020 [3]. Based on projected changes in population growth and aging—and assuming that the overall cancer incidence remains unchanged—more than 35 million new cancer cases are expected to occur by 2050, representing a 77% increase compared to 2022 [4]. Recent estimates provided by the Canadian and American Cancer Societies indicate that over 40% of individuals will be diagnosed with cancer during their lifetime [5]. In Iran, in addition to substantial geographic variation in the incidence rates of various cancer types across different provinces, the number of new cancer cases is projected to increase by more than 40% between 2016 and 2025, primarily due to changes in risk factors and population structure [3].

Hematologic malignancies may arise from abnormal differentiation of hematopoietic stem cells and affect the development of red blood cells, white blood cells, and platelets [6]. The three common categories of hematologic malignancies include leukemia, lymphoma, and plasma cell neoplasms (multiple myeloma) [7]. Leukemia and multiple myeloma are malignancies of blood tissues or cells that involve the bone marrow, leading to irregular proliferation of bone marrow progenitor cells [8]. These malignancies ranked 13th and 21st, respectively, among the most commonly diagnosed cancers in 2022 [4]. Lymphoma is a malignancy of the lymphatic system and is classified into two types: Hodgkin lymphoma and non‐Hodgkin lymphoma [9].

Cancer patients face diverse health challenges due to the disease’s nature and varied symptoms, requiring specialized, individualized care [10–12]. Nurses are responsible for safe, comprehensive care across healthcare settings, with quality improvement a priority. Oncology nurses are crucial in delivering evidence‐based care that addresses patients’ clinical, supportive, communicative, and spiritual needs. Patients’ perceptions define the quality of oncology nursing care [13, 14].

The nursing care delivery model significantly impacts care quality [15–17]. Organizations should adopt models aligned with their mission and resources, with patient‐centered approaches being particularly effective [16, 18]. A number of models include functional, individual (case method), team, and primary nursing [18]. These approaches differ in how responsibility for patients is allocated, how nurses collaborate, and how continuity of care is maintained across shifts [15–17].

The case method involves a nurse assuming full responsibility for a patient group during a shift [18, 19]. The case method is based on assigning a nurse direct responsibility for the care of a defined group of patients during a particular shift [18, 19]. By concentrating responsibility for a patient’s care within an individual nurse during the assigned shift, this model may facilitate individualized attention and direct communication with patients [19]. At the same time, because nursing responsibility may change from one shift to another, maintaining consistency in care requires effective communication and transfer of information among nurses. When communication during these transitions is insufficient, differences in care planning or implementation may occur between successive shifts [18]. Thus, the case method provides a clear framework for individual responsibility, while its effectiveness may also depend on the quality of communication and coordination within the nursing team.

The term primary nursing represents a different approach to organizing nursing responsibilities, with greater emphasis on continuity and individual accountability [20]. The model was introduced in the United States by Marie Manthey in 1968 and was developed around the concept of assigning a primary nurse responsibility for coordinating a patient’s nursing care throughout hospitalization [18, 21]. Depending on the care arrangement, the primary nurse may plan and evaluate care directly or establish the care plan according to which other nurses carry out delegated activities. Consequently, primary nursing seeks to establish a more continuous framework for the planning and delivery of care and to maintain an ongoing professional relationship between nurses and patients [16, 18, 21].

Continuity of care is considered an important dimension of healthcare delivery and may affect patients’ experiences and outcomes. Patients have reported valuing continuity in their care [22], and previous research has identified associations between continuity of communication and patient‐related outcomes [23, 24]. In particular, poorer continuity of communication has been associated with lower cognitive functioning, whereas better continuity has been related to greater satisfaction with the overall hospital experience [25]. These findings highlight the potential importance of continuity within nursing care; however, they do not in themselves establish that a particular nursing care delivery model is superior to another.

Despite the long‐standing use of primary nursing in different healthcare environments, the empirical evidence concerning its effectiveness remains limited. Much of the available literature was published several decades ago, and the findings have not provided a consistent conclusion regarding the advantages of primary nursing [21]. In addition, claims concerning the benefits of primary nursing compared with alternative models have often been based on descriptive or narrative evidence rather than robust comparative research [18]. Therefore, the extent to which the organizational characteristics of primary nursing translate into measurable improvements in nursing care quality remains uncertain.

Despite the long‐standing use of primary nursing in different healthcare environments, the empirical evidence concerning its effectiveness remains limited. Much of the available literature was published several decades ago, and the findings have not provided a consistent conclusion regarding the advantages of primary nursing [20]. In addition, claims concerning the benefits of primary nursing compared with alternative models have often been based on descriptive or narrative evidence rather than robust comparative research [17]. Therefore, the extent to which the organizational characteristics of primary nursing translate into measurable improvements in nursing care quality remains uncertain.

The potential relationship between nursing care delivery models and outcomes such as care quality, patient satisfaction, nurse satisfaction, and clinical results has been examined in previous studies [19, 21, 22, 25–30]. Nevertheless, the available evidence remains insufficient to determine whether one model consistently provides better nursing care across different clinical settings and patient populations. In particular, comparatively little research has focused on the quality of nursing care delivered to patients with hematological malignancies. These patients frequently require complex and prolonged treatment, close clinical monitoring, coordination of multiple aspects of care, and adaptation of nursing interventions to changes in their clinical condition. Consequently, the organization of nursing responsibilities may be particularly relevant to the quality and consistency of care provided to this population.

Although both the case and primary nursing approaches provide mechanisms for organizing nursing responsibilities, their respective effects on the quality of nursing care may differ according to the clinical context and the way each model is implemented. The limited evidence available in oncology settings, together with the lack of sufficient comparative research among patients with hematological malignancies, represents an important gap in the literature. Addressing this gap may provide evidence to support decisions regarding the organization of nursing care in specialized hematology settings. Therefore, this study aimed to compare the quality of nursing care provided through the Primary Method and Case Method Approaches among patients with hematological malignancies.

## 2. Method

This study was a descriptive‐comparative investigation conducted to evaluate and compare the quality of oncology nursing care using two care delivery models—team‐based primary nursing and the case method—in patients diagnosed with hematologic malignancies. Data were collected between October 2024 and March 2025 using the “Quality of Oncology Nursing Care Scale (QONCS),” developed by Andreas Charalambous in 2014 [11].

### 2.1. Setting

The study was conducted in the hematology and bone marrow transplantation (BMT) units of Ayatollah Taleghani Hospital, Shahid Beheshti University of Medical Sciences, Tehran, Iran. Between October 2024 and March 2025, 231 patients admitted to the hematology ward and 253 patients admitted to the BMT unit.

The number of patients in each unit, the patient‐to‐nurse ratio per shift, patients’ need for supportive care and assistance with self‐care, reverse isolation conditions during transplantation, and the organizational culture of each unit may influence nurses’ workload, job satisfaction, and work motivation, ultimately affecting the quality of nursing care and may, therefore, act as potential confounding variables. Therefore, these factors, together with the demographic characteristics of patients and nurses, were examined and compared between the hematology and BMT units.

The hematology ward, with 22 beds, was staffed by 10 nurses (including a head nurse) using the case method. The BMT unit, with 20 beds, employed 14 nurses (including a head nurse and an apheresis specialist) in three four‐member teams following a team‐based primary nursing model. The head nurses of the two units were not directly responsible for providing patient care. In the hematology unit, 9 nurses were responsible for direct patient care, whereas 12 nurses were responsible for patient care in the BMT unit. During the study period, nurses remained assigned to their respective units, and no routine reassignment or rotation of nurses occurred between the BMT, hematology, or other hospital units.

In the BMT unit, these 12 nurses were organized into three teams, with each team consisting of a team leader and other nursing staff. The team leader was responsible for following up on patients’ care needs, coordinating care activities, and communicating with other members of the team. The roles and responsibilities of nurses, patient assignment procedures, accountability, and mechanisms for continuity of care were defined according to the unit’s established care processes. In the hematology unit, care was provided according to the usual nursing care model of the unit. During data collection, the implementation of the respective care models was monitored as part of routine clinical practice.

The mean (standard deviation [SD]) years of professional experience among nurses in the hematology ward was 10.40 (SD = 7.42) years, compared to 8.28 (SD = 7.94) years in the BMT unit, with no statistically significant difference between the groups (*t* = 0.66, df = 22, *p* = 0.52). Similarly, the mean work experience in the respective department was 6.60 ± 6.73 years in the hematology ward and 5.86 ± 5.26 years in the BMT unit (*t* = 0.30, df = 22, *p* = 0.76). A statistically significant difference was observed in gender distribution, with all nurses in the BMT unit being female compared with 60% in the hematology ward (Fisher’s exact test, *p* = 0.02). No significant differences were found in marital status (*p* = 0.08) or type of work shift (*p* = 1.00).

Regarding workload, the mean number of patients per day was significantly higher in the BMT unit than that in the hematology ward (14.07 ± 1.51 vs. 12.67 ± 1.06; Welch’s *t* = 4.16, df = 52.09, *p* < 0.001). Similarly, the mean number of patients per nurse per shift was significantly higher in the BMT unit than in the hematology ward (4.69 ± 0.50 vs. 4.22 ± 0.35; paired *t*(29) = 3.58, *p* = 0.001). These findings indicate a greater patient workload for nurses in the BMT unit. Table 1 shows demographic, occupational characteristics, and patient workload of nurses by ward.

**TABLE 1:** Demographic, occupational characteristics, and patient workload of nurses by ward.

Variable	Category/indicator	Hematology (*n* = 10)	BMT (*n* = 14)	Test result, *p* value
Gender	Female, *n* (%)	6 (60.0)	14 (100)	Fisher’s exact test, *p* = 0.02
	Male, *n* (%)	4 (40.0)	0 (0)	

Marital status	Single, *n* (%)	5 (50.0)	5 (35.7)	Continuity correction, *p* = 0.08
	Married, *n* (%)	5 (50.0)	9 (64.3)	

Type of work shift	Fixed shift, *n* (%)	3 (30.0)	4 (28.6)	Fisher’s exact test, *p* = 1.00
	Rotating shift, *n* (%)	7 (70.0)	10 (71.4)	

Years of professional work experience	Mean ± SD	10.40 ± 7.42	8.28 ± 7.94	*t* = 0.66, df = 22, *p* = 0.52
Years of work experience in the department	Mean ± SD	6.60 ± 6.73	5.86 ± 5.26	*t* = 0.30, df = 22, *p* = 0.76
Number of patients per day	Mean ± SD	12.67 ± 1.06	14.07 ± 1.51	Welch’s *t* = 4.16, df = 52.09, *p* < 0.001
Number of patients per nurse per shift	Mean ± SD	4.22 ± 0.35	4.69 ± 0.50	*t* = 3.58, df = 29 *p* = 0.001

*Note:* Percentages are based on the total number of nurses in each ward. The number of patients per day was compared using Welch’s independent‐samples *t*‐test, whereas the number of patients per nurse per shift was compared using a paired‐samples *t*‐test based on 30 matched shifts.

Abbreviations: BMT = bone marrow transplantation, SD = standard deviation.

### 2.2. Participants

The study population included patients over 18 years old with hematological malignancies (leukemia, lymphoma, or multiple myeloma) and their family who were hospitalized for more than 48 h in the hematology and BMT units of Ayatollah Taleghani Hospital, affiliated with Shahid Beheshti University of Medical Sciences, and were able to read. All patients had confirmed diagnoses from a specialist physician.

### 2.3. Research Tool

Participants completed a questionnaire consisting of two sections. The first section collected patients’ demographic and clinical characteristics, including age, sex, marital status, educational level, occupation, hospital ward, type and reason for hospitalization, length and frequency of hospitalization, underlying diseases, treatment‐related complications, health insurance status, access to home care, self‐care ability, and perceived adequacy of income. Information on nurses’ demographic and professional characteristics, including sex, marital status, work experience, experience in the current ward, and work shift, was also collected.

The second section comprised the QONCS, originally developed by Charalambous et al. [11]. The original 35‐item instrument evaluates patients’ perceptions of the quality of comprehensive oncology nursing care using a five‐point Likert response scale, with higher scores indicating better perceived quality of nursing care.

For use in the Iranian context, the QONCS underwent forward translation, backtranslation, and cultural adaptation and expert assessment of face and content validity. Following exploratory and confirmatory factor analyses (CFAs), a preliminary 29‐item, three‐factor structure was identified, comprising compassionate care, spiritual care, and respect for patients’ beliefs. Although model‐fit indices were below recommended thresholds, the instrument demonstrated high internal consistency (Cronbach’s alpha = 0.97 for the total scale). Therefore, the preliminary validated Persian version was used to assess patients’ perceived quality of oncology nursing care in the present study [31].

### 2.4. Comparison of the Factor Structure of the Instrument Between the Hematology and BMT Units

To examine the applicability of the instrument in the hematology and BMT units, the results of the three‐factor CFA models were compared between the two groups. In both groups, the instrument comprised 29 items distributed across three latent factors, and the overall pattern of relationships between the items and their corresponding factors showed relative similarity.

The model fit indices indicated that the chi‐square‐to‐degrees‐of‐freedom ratio was 2.560 in the hematology group and 2.176 in the BMT group. The CFI values were 0.782 and 0.793, respectively, while the corresponding TLI values were 0.746 and 0.760. The RMSEA values were 0.135 for hematology and 0.117 for BMT. Although some fit indices were comparatively more favorable in the BMT group, the overall fit indices indicated limitations in the fit of the proposed model to the observed data in both groups. Therefore, the findings suggest a degree of similarity in the factor structure across the two units; however, they do not provide sufficient evidence to establish an adequate overall model fit in either group (Supporting 1).

A comparison of the standardized factor loadings showed that the loadings of many items belonging to Factors 1 and 2 were higher in the hematology group. In contrast, several items associated with Factor 3, including Q27, Q28, Q29, and Q30, had higher loadings in the BMT group. Despite these descriptive differences, the overall pattern of item allocation to the three factors was consistent across the two groups. Furthermore, the correlation between Factors 1 and 3 was high in both groups, reaching 0.956 in hematology and 0.876 in BMT. This finding highlights the need to further examine the conceptual distinctiveness of these two factors (Supporting 1).

Overall, the available findings indicate a relative similarity in the factor structure of the instrument between the hematology and BMT units. The observed differences in model fit indices and factor loadings, however, cannot by themselves establish that the underlying measurement structures differ between the groups. Nevertheless, given the limited sample size, the suboptimal model fit in both groups, and the absence of a formal measurement invariance analysis, the equality of measurement parameters and the comparability of instrument scores across the two units cannot be established with sufficient confidence. Accordingly, these findings should be interpreted as preliminary.

### 2.5. Sample Size

The sample size was obtained using the following formula in each group:

(1) n≥2zα/2+zβ2 σ2 μ1−μ2.

The required sample size was calculated using the following formula for each group. The calculation was based on a two‐sided significance level of 0.05 (*α* = 0.05), a statistical power of 90% (1−*β* = 0.90), an expected standardized effect size of 0.50 (Cohen’s d), and an estimated SD (*σ*) of 1.28. Based on these assumptions, the minimum required sample size was 84 participants per group (168 participants in total). To account for an anticipated 4% dropout rate, the target sample size was increased to 86 participants per group.

Between October 2024 and March 2025, 86 eligible patients admitted to the hematology ward (of 231 admissions) and 87 eligible patients admitted to the BMT unit (of 253 admissions) were enrolled in the study after meeting the predefined inclusion criteria.

### 2.6. Data Collection

Following ethics committee approval, the researcher collected data at designated centers. All patients provided informed consent after being informed about the study’s purpose and confidentiality before completing the questionnaire. All questionnaires were completed in the presence of the researcher. For patients with severe illness or treatment‐related complications who were unable to complete the questionnaires independently, alternative data collection procedures were used. In the hematology unit, family caregivers assisted in completing the questionnaires on behalf of patients who were unable to respond independently. Written informed consent was obtained from the patients and their family members before participation in the study. Efforts were made to standardize the questionnaire administration procedures across the two units and ensure consistency in data collection. The datasets of the current study are available as Supporting file 2.

### 2.7. Data Analysis

Data were analyzed using SPSS software Version 20 (IBM Corp., Armonk, NY) and Psychometrica [32], with statistical significance set at *p* < 0.05. Descriptive statistics (*N*, %, mean, SD) summarized demographic characteristics. The Kolmogorov–Smirnov test assessed the normality of continuous variables (age, hospitalization duration, and hospital admission frequency). Group homogeneity was evaluated using independent *t*‐tests for normally distributed continuous variables (age and hospitalization duration); Mann–Whitney *U* test for nonnormally distributed continuous variables (hospital admission frequency); and chi‐square and Fisher’s exact tests for categorical variables. An independent *t*‐test compared perceived oncology nursing care quality between groups.

### 2.8. Handling of Missing Data and Incomplete Questionnaires

Missing data were handled using an analysis‐by‐analysis exclusion approach (available‐case analysis). Subscale and total scores were calculated according to the questionnaire’s scoring guidelines, which permit score calculation when some item responses are missing. Participants with valid scores for the relevant outcomes were included in the corresponding analyses, and the number of participants retained for each analysis is reported alongside the respective results. As a sensitivity analysis, the relevant comparisons were repeated using listwise deletion to assess the robustness of the findings to the missing‐data handling approach. For regression analyses, questionnaires with incomplete data on the variables required for the respective models were excluded using listwise deletion, and the number of complete cases retained in each model was documented. Consequently, the analytical sample size varied across analyses according to data completeness, scoring requirements, and the variables included in each model.

## 3. Results

The study included 173 hospitalized patients from the hematology (86 persons) and BMT (87 persons). The participants were 59.5% male (*n* = 103) and 40.5% female (*n* = 70). The mean age was 50.38 (SD = 16.67) years for hematology patients and 47.54 (SD = 13.59) years for BMT patients. Table 2 presents a comparative overview of the demographic characteristics of participants in the hematology and BMT units.

**TABLE 2:** Demographic characteristics of the participants in the Hematology and BMT units.

		Group/ward				Test result
		Hematology		BMT		
		Count	*N* %	Count	*N* %	
Gender	Female	38	22.0%	32	18.5%	*X* ^2^ = 0.98 df = 1 *p* = 0.32
	Male	48	27.7%	55	31.8%	
	Total	86	50%	87	50%	

Marriage	Single	17	9.8%	19	11.0%	Fisher = 4.710 *p* = 0.106
	Married	62	35.8%	67	38.7%	
	Widowed	7	4.0%	1	0.6%	
	Total	86	49.7%	87	50.3%	

Education	Elementary	28	16.2%	10	5.8%	*X* ^2^ = 16.61 df = 3 *p* = 0.001
	High school	35	20.2%	33	19.1%	
	Undergraduate	19	11.0%	30	17.3%	
	Graduate	4	2.3%	14	8.1%	
	Total	86	49.7%	87	50.3%	

Underlying disease	No	72	41.6%	71	41.0%	*X* ^2^ = 0.13 df = 1 *p* = 0.71
	Yes	14	8.1%	16	9.2%	
	Total	86	50%	87	50%	

Insurance	No	1	0.6%	0	0.0%	Fisher’s Exact Test *p* = 0.50>
	Yes	85	49.1%	87	50.3%	
	Total	86	50%	87	50%	

Complication	No	54	31.4%	46	26.7%	*X* ^2^ = 1.53 df = 1 *p* = 0.22
	Yes	32	18.6%	40	23.3%	
	Total	86	50%	86	50%	

	**Group/ward**	**N**	**Mean**	**Standard of deviation**		**Test result**

Age	Hematology	86	50.38	16.67		df = 171 *p* = 0.22
	BMT	87	47.54	13.59		
	Total	173	48.95	15.22		

The length of hospitalization	Hematology	86	11.80	9.40		df = 171 *p* = 0.842
	BMT	87	11.53	8.63		
	Total	173	11.66	9.00		

### 3.1. Findings of Quality of Oncology Nursing Care

Independent‐samples *t*‐tests were used to compare oncology nursing care scores between the hematology and BMT units. The assumption of homogeneity of variances was assessed using Levene’s test. Equal variances were assumed for spiritual care (*p* = 0.381); therefore, the conventional *t*‐test was applied, resulting in 135 degrees of freedom. In contrast, the homogeneity of variance assumption was violated for compassionate care, respect for patients’ beliefs, and overall quality of oncology nursing care (*p* < 0.05); consequently, Welch’s *t*‐test was used for these comparisons. This resulted in adjusted, noninteger degrees of freedom (124.82, 120.32, and 104.50, respectively).

As presented in Table 3, the overall quality of oncology nursing care was significantly higher in the BMT unit than that in the hematology unit (121.69 ± 16.97 vs. 110.53 ± 22.88), *t*(104.50) = −3.03, *p* = 0.003, and Cohen’s *d* = 0.56. Compassionate care was also higher in the BMT unit (59.25 ± 6.97 vs. 50.66 ± 10.96), *t*(124.82) = −5.85, *p* < 0.001, and Cohen’s *d* = 0.95, as was respect for patients’ beliefs (31.44 ± 4.53 vs. 26.97 ± 6.28), *t*(120.32) = −4.95, *p* < 0.001, and Cohen’s *d* = 0.83. No significant between‐group difference was observed for spiritual care (31.74 ± 8.08 vs. 30.65 ± 7.60), *t*(135) = −0.81, *p* = 0.419, Cohen’s *d* = 0.14.

**TABLE 3:** The comparative results of oncology nursing care quality across the two units.

Variable	Group/ward	*N*	Mean (SD)	Test result	Effect size (Cohen’s *d*)	Statistically significant
Compassionate care	Hematology	76	50.66 (10.96)	*t* = −5.846, df = 124.824, *p* < 0.001	0.95	Yes
	BMT	84	59.25 (6.97)			
	Total	160	55.17 (10.03)			

Spiritual care	Hematology	72	30.65 (7.60)	*t* = −0.811, df = 135, *p* = 0.419	0.14	No
	BMT	65	31.74 (8.08)			
	Total	137	31.17 (7.82)			

Respect for patients’ beliefs	Hematology	69	26.97 (6.28)	*t* = −4.951, df = 120.317, *p* < 0.001	0.83	Yes
	BMT	85	31.44 (4.53)			
	Total	154	29.44 (5.81)			

Quality of oncology nursing care	Hematology	58	110.53 (22.88)	*t* = −3.033, df = 104.498, *p* = 0.003	0.56	Yes
	BMT	64	121.69 (16.97)			
	Total	122	116.39 (20.68)			

*Note:* Missing data were handled using the analysis‐by‐analysis exclusion method (available‐case analysis), whereby cases with missing values were excluded separately for each analysis. Data are presented as mean (standard deviation [SD]). Between‐group comparisons were conducted using independent‐samples *t*‐tests. Welch’s *t*‐test was used for compassionate care, respect for patients’ beliefs, and quality of oncology nursing care because the assumption of equal variances was violated. The conventional independent‐samples *t*‐test assuming equal variances was used for spiritual care. Effect sizes were calculated using Cohen’s d and interpreted as small (0.20), medium (0.50), and large (0.80). A *p* value of less than 0.05 was considered statistically significant.

Abbreviation: BMT = bone marrow transplantation.

Sensitivity analyses using listwise deletion yielded a similar pattern of statistical significance to the primary available‐case analyses. Between‐group differences remained statistically significant for compassionate care, respect for patients’ beliefs, and the total score, whereas the difference in spiritual care, remained no statistically significant. However, the estimated mean differences for compassionate care and respect for patients’ beliefs varied between the two approaches, indicating some sensitivity of the magnitude of the observed differences to the missing‐data handling method.

### 3.2. Multivariable Analysis

A generalized linear model was conducted to investigate whether ward assignment was related to the quality of oncology nursing care after accounting for selected demographic and clinical characteristics. The results of the generalized linear model examining factors associated with the quality of oncology nursing care are presented in Table 4. The analysis included 122 participants with complete observations, representing 70.5% of the 173 records available in the dataset. The overall mean score for quality of oncology nursing care was 116.39 (SD = 20.68), with observed scores ranging from 49 to 145.

**TABLE 4:** Generalized linear model examining factors associated with quality of oncology nursing care.

Predictor	B	SE	Wald *χ* ^2^	*p*	95% CI for B
**Ward/group (hematology vs. BMT)**	**−9.472**	3.858	**6.027**	**0.014**	**[−17.034, −1.910]**
Education	−0.276	2.411	0.013	0.909	[−5.001, 4.449]
Age	−0.179	0.131	1.870	0.171	[−0.436, 0.078]
Gender	−3.574	3.716	0.925	0.336	[−10.858, 3.710]
Disease	−0.745	1.730	0.186	0.667	[−4.135, 2.645]
Hospitalization duration	−0.294	0.197	2.237	0.135	[−0.680, 0.091]
**Salary**	**9.455**	4.110	**5.293**	**0.021**	**[1.400, 17.509]**

*Model fit*					
Omnibus likelihood‐ratio *X* ^2^(7)			**21.335**	**0.003**	
AIC					**1081.007**
*N*					**122**

*Note: B* = unstandardized regression coefficient; Wald *χ* ^2^ = Wald chi‐square statistic. BMT was considered the reference category for ward/group. The generalized linear model was fitted using a normal distribution with an identity link. Bold values indicate statistically significant associations (*p* < 0.05).

Abbreviations: AIC = Akaike information criterion, CI = confidence interval, SE = standard error.

The fitted multivariable model demonstrated a statistically significant overall effect (likelihood ratio *X* ^2^(7) = 21.335, *p* = 0.003). Ward assignment continued to show a significant relationship with the quality‐of‐care score after controlling for education, age, gender, disease, hospitalization duration, and salary (Wald *X* ^2^(1) = 6.027, *p* = 0.014). Taking the BMT ward as the reference category, the estimated score for nurses in the hematology ward was 9.47 points lower (*B* = −9.472, SE = 3.858, 95% CI [−17.034, −1.910], *p* = 0.014).

Of the variables included in the adjusted model, salary was the only covariate other than ward assignment that showed a statistically significant association with the outcome (*B* = 9.455, SE = 4.110, 95% CI [1.400, 17.509], *p* = 0.021). No statistically significant associations were observed for education (*B* = −0.276, *p* = 0.909), age (*B* = −0.179, *p* = 0.171), gender (*B* = −3.574, *p* = 0.336), disease (*B* = −0.745, *p* = 0.667), or hospitalization duration (*B* = −0.294, *p* = 0.135). The Akaike information criterion for the adjusted model was 1081.007.

## 4. Discussion

The present study aimed to compare the quality of oncology nursing care delivered through team‐based primary nursing and case‐based methods among patients with hematologic malignancies. A total of 173 hospitalized patients from the hematology and BMT units of Ayatollah Taleghani Hospital participated in the study. The findings revealed that the overall mean score for oncology nursing care quality was significantly higher in the BMT unit, where care was delivered using a team‐based primary nursing model. This approach also demonstrated superior performance in specific dimensions of care quality, particularly in the domains of “compassionate care, and respect for patients’ beliefs.”

According to Charalambous et al., individualized care is essential for achieving high‐quality oncology nursing [11]. Given that the primary nursing model offers an innovative, personalized, and patient‐centered approach to care delivery [16, 24], the present findings may align with those of Charalambous et al. in the domains of “Support and Confirmation” and “Respect,” while potentially diverging in areas such as “Spiritual Care,” “Sense of Belonging,” and “Being Valued” [11]. In this study, the significantly higher overall quality score observed in the BMT unit may be consistent with the findings of Tonkikh et al. [24], who reported that the primary nursing model contributes to achieving higher quality standards in care delivery from the nurses’ perspective. Similarly, the results correspond with those of Ventura‐Silva et al. [21], in which most patients reported high overall care quality. Conversely, the findings of Freitas et al. suggested that primary nursing may be less effective than anticipated, highlighting the need for further investigation into contextual and implementation factors [33].

The low mean scores in “Spiritual Caring,” with no significant differences between units, mirror findings by Freitas et al., where the lowest scores were linked to spiritual and social needs [34]. They highlighted the challenge for nursing to broaden its scope to include patients’ emotional, spiritual, and psychological needs [34]. Consistent with other international psychometric evaluations [35–37] using similar instruments, this study also found low average scores in “Spiritual Care.” This suggests a widespread tendency for nurses to overlook patients’ spiritual and religious needs, which may be exacerbated by high workloads and nursing shortages in oncology units, thereby limiting patient interaction and spiritual care [11, 31, 35].

This deficiency may also arise from nurses’ hesitation to discuss spiritual and religious matters due to cultural differences or perceived inadequacy, often due to insufficient training [10, 11, 35]. Considering the integral role of spiritual care in comprehensive oncology care [11, 36], it is crucial to prioritize this aspect in oncology nursing practice. Given that patient satisfaction is considered the most critical indicator of care quality and reflects the outcomes of healthcare services, the quality and adequacy of medical care can be assessed through the perspectives and satisfaction levels of patients and their families [34, 38, 39]. Accordingly, the findings of the present study are consistent with those of previous research, suggesting that the primary nursing care model may contribute to enhanced care quality.

While Roshandel et al. and Chen et al. found shorter hospital stays with primary nursing [3, 26], this study found no significant difference in the length of stay between units, nor any relationship between the length of stay and quality of care. This may be due to the longer treatment times required in the bone marrow transplant unit. Further research is needed to evaluate the impact of care delivery models on hospitalization duration for patients with similar treatment needs. Havaei et al. found that team nursing, compared to case‐based models, led to more missed tasks and adverse outcomes in acute care, suggesting compromised continuity and accountability [39]. Similarly, Freitas et al. found no significant improvement in care quality with primary nursing compared to functional or team‐based models, suggesting that structural changes alone may not improve patient outcomes [33].

In the present study, no statistically significant differences were observed in disease‐related complications between the two nursing models. However, a notable disparity in hospitalization frequency was identified, with the Hematology unit exhibiting a significantly higher rate of admissions. This may reflect the inherent complexity and intensity of treatment protocols in hematologic malignancies, which are often accompanied by multifaceted complications. Nevertheless, the absence of a statistically significant correlation between care quality and hospitalization frequency suggests that adverse events may be intrinsic to the treatment process itself, rather than attributable to the specific model of care delivery. This finding highlights the need for a more nuanced understanding of care outcomes in specialized clinical contexts.

The higher quality of nursing care observed in the BMT unit, despite its greater patient workload, may reflect differences in the organization and delivery of nursing care, in addition to patient numbers. Patients in BMT units require specialized care, continuous monitoring, strict adherence to therapeutic and infection‐prevention protocols, and close coordination among healthcare professionals. In this context, effective teamwork, appropriate task allocation, and communication among nurses may help maintain care standards. Previous research has highlighted the importance of collaboration within nursing care delivery models and the influence of organizational and staffing factors on care quality and patient safety [17, 18, 39].

The team‐based nursing care model may also facilitate information sharing and the development of a more comprehensive understanding of patients’ physical, psychological, emotional, social, and family‐related needs. Such collaboration may support coordinated follow‐up and promote compassionate, patient‐centered, and holistic care. In contrast, under the case method, a nurse is responsible for assigned patients during a particular shift, with continuity depending partly on accurate documentation and effective handover. Research comparing nursing care delivery models has highlighted the importance of care organization and the way responsibilities are allocated in shaping patients’ care experiences [18, 19, 28, 29]. Nevertheless, the potential advantages of team‐based care depend on effective coordination, clear accountability, and appropriate staffing. Havaei et al. found that team‐based care was not consistently associated with better outcomes, emphasizing the importance of effective teamwork and the clinical context in which a model is implemented [39].

Accordingly, the higher care quality observed in the BMT unit should not be interpreted as evidence that workload is unimportant or that the team‐based model alone explains the observed difference. Patient acuity and clinical complexity, nurse‐to‐patient ratios, staffing adequacy, nurses’ professional experience, shift patterns, and organizational characteristics may also have contributed. Given the observational and cross‐sectional design, the relative contributions of these factors cannot be determined. Future studies should investigate the relationship between nursing care models, workload, and care quality while accounting for relevant patient‐level, nurse‐level, and organizational factors.

The findings also indicated a relatively similar three‐factor structure of the QONCS across the hematology and BMT units. This finding provides preliminary support for the potential applicability of the instrument in both settings. The original QONCS development study and subsequent cross‐cultural validation studies have demonstrated the importance of evaluating the psychometric properties of this instrument in different populations and cultural contexts [11, 14, 31, 36, 37]. However, differences in factor loadings and model‐fit indices suggest that relationships between individual items and their corresponding factors may vary across clinical settings. The limited sample size may also have reduced the statistical power to detect differences. Therefore, the absence of sufficient evidence for a difference should not be interpreted as a proof of equivalence. The present findings are insufficient to establish measurement invariance or confirm that scores can be directly compared across the two units. Further research involving larger samples, improved model fit, and formal measurement invariance testing is needed to establish the stability and comparability of the instrument across clinical environments.

An additional methodological limitation concerns differences in data collection procedures between the two units. In the hematology unit, approximately 70% of the questionnaires were completed by family caregivers in the presence of the patients and the researcher, whereas data in the BMT unit were collected through structured, researcher‐administered interviews. Although these approaches facilitated data collection, differences in the respondent type and administration mode may have influenced responses and limited comparability between the units. The presence of patients during caregiver‐assisted completion does not necessarily ensure that caregivers’ responses fully reflect patients’ own perceptions. This issue is particularly relevant to subjective dimensions of nursing care, including emotional support, respect, and spiritual care. Consequently, the observed differences may reflect not only variations in patients’ care experiences but also potential measurement bias. Future studies should standardize data collection procedures across settings, prioritize direct patient‐reported outcomes whenever clinically feasible, and systematically document the respondent type to improve measurement consistency and facilitate sensitivity analyses.

Finally, because of the observational and cross‐sectional design, causal conclusions cannot be drawn regarding the effect of nursing care delivery models on care quality. Future research should employ prospective or intervention‐based designs, including randomized controlled trials where feasible, to examine the effects of different nursing care models on care quality and workload. Such studies should account for patient acuity and clinical complexity, nurse‐to‐patient ratios, staffing adequacy, nurses’ professional experience and expertise, shift patterns, teamwork, and organizational characteristics. Evaluating strategies such as optimizing staffing and skill mix, balancing workload distribution, strengthening team collaboration, and implementing structured handover protocols may help identify effective approaches to improving nursing care quality and safety. This evidence could clarify the contribution of different nursing care models to continuity of care, comprehensive assessment of patients’ needs, and quality improvement in specialized hematology and BMT settings.

### 4.1. Practical Implications for Nurse Managers and Policymakers

The findings of this study have practical implications for nursing management in Hematology and BMT settings. Nurse managers may consider care delivery approaches that promote continuity of care, clear professional responsibilities, effective communication, and a more comprehensive understanding of patients’ individual needs and experiences. Such approaches should support holistic, patient‐centered care by integrating physical, psychosocial, emotional, and spiritual needs into routine nursing practice. Particular attention is warranted for spiritual and psychosocial care, as these dimensions received the lowest ratings in the present study, irrespective of the care model. Organizational strategies should also consider staffing adequacy, patient‐to‐nurse ratios, workload, nurses’ professional competencies, and the clinical and environmental demands of each unit when planning nursing services. Although the findings suggest differences in perceived nursing care quality between the two settings, these differences cannot be attributed solely to the nursing care models because other patient‐, nurse‐, and unit‐level factors may also have contributed. Therefore, nursing leaders should consider care models that facilitate continuity, individualized care, and meaningful nurse–patient relationships while evaluating their implementation in the local clinical context. Further multicenter and prospective studies are needed to examine whether these approaches can consistently improve patients’ experiences and the quality of oncology nursing care across different healthcare settings.

## 5. Conclusion

The quality of oncology nursing care reported by patients was higher in the BMT unit, where team‐based primary nursing was implemented, than that in the hematology unit, where the case method was used. Team‐based primary nursing may be associated with higher quality of car score in hematology–oncology units than the case method, particularly in patient‐centered aspects like affirmation, emotional support, recognition of individual value, and respectful interaction. These findings suggest that the organization and delivery of nursing care may contribute to promoting patient‐centered care and improving the care experience of patients with hematologic malignancies.

However, the findings should be interpreted with caution because of the study design and the potential influence of contextual and organizational factors, including patient complexity, staffing levels, nurses’ experience and expertise, workload, and team functioning. Therefore, the observed differences cannot be interpreted as definitive evidence of a causal effect of the nursing care model. Further experimental and quasiexperimental studies, preferably with larger and more diverse samples and appropriate control of potential confounding factors, are needed to compare different nursing care models and determine their effects on nursing care quality, patient satisfaction, patient safety, and nurses’ workload. Such evidence could support the development of evidence‐based strategies and policies for optimizing nursing care delivery in hematology–oncology and other specialized clinical settings.

### 5.1. Limitations

This study has several limitations that should be considered when interpreting the findings. First, the single‐center design and the inclusion of patients from hematology and BMT units in one hospital in Iran may limit the generalizability of the findings to other hospitals, healthcare systems, and patient populations. Differences in organizational structures, staffing patterns, nurse‐to‐patient ratios, institutional policies, available resources, and models of nursing care may influence the quality of care across settings. Therefore, the findings may not fully represent the broader context of hematology–oncology nursing care in Iran or other healthcare systems.

Second, the relatively limited sample size may have reduced the statistical power to detect smaller differences between groups and may have limited the precision of the estimated effects. Future studies with larger samples and participation from multiple centers are needed to improve statistical power and external validity.

Third, given the observational and cross‐sectional design of the study, causal relationships between the nursing care model and the quality of nursing care cannot be established. Although differences in care quality were observed across study settings, these differences may have been influenced by potential confounding factors, including patient acuity and clinical complexity, nurses’ professional experience and expertise, staffing adequacy, workload, shift patterns, teamwork, and organizational characteristics. Future studies should employ intervention‐based or longitudinal designs and appropriately control for potential confounding factors at the patient, nurse, and unit levels to provide more robust evidence regarding the relationship between nursing care models and the quality of nursing care.

Fourth, restricted visitation in the BMT unit, which was required because of protective isolation and infection‐prevention measures, may have influenced patients’ perceptions of social interaction and their “sense of belonging.” Consequently, responses in this domain may not be directly comparable with those of patients receiving care in less restrictive environments.

Fifth, the relatively small sample size in the hematology and BMT groups may have limited the statistical power to detect differences in the factor structure and measurement parameters between the two groups. Consequently, the observed similarity in the factor loadings and overall factor structure should be interpreted cautiously and cannot be regarded as definitive evidence of measurement equivalence. Second, the model fit indices indicated suboptimal fit in both groups, suggesting that further evaluation and potential refinement of the proposed three‐factor model may be necessary.

Finally, differences in data collection methods between the two study units may have introduced measurement bias and limited the comparability of responses, particularly regarding subjective dimensions of nursing care, such as emotional support, respect, interpersonal interactions, and sense of belonging. In the hematology unit, approximately 70% of the questionnaires were completed by family caregivers in the presence of the patients and the researcher, whereas data in the BMT unit were collected through structured, researcher‐administered interviews. In addition, for severely ill or physically compromised patients who were unable to complete the questionnaires independently, assistance was provided by family caregivers or the researcher. Although these approaches facilitated data collection, proxy responses and differences in administration methods may have influenced participants’ responses, as caregivers’ or researchers’ perceptions may differ from those of patients. Furthermore, the respondent type was not systematically documented at the individual questionnaire level, preventing the reliable identification of all proxy‐completed questionnaires and precluding sensitivity analyses excluding proxy responses. Therefore, the potential effects of the respondent type and data collection mode on the findings cannot be ruled out. Future studies should standardize questionnaire administration procedures across study settings, systematically document the respondent type, and prioritize direct patient‐reported outcomes whenever clinically feasible to enhance measurement consistency and enable sensitivity analyses.

**Nomenclature**
QONCS: Quality of Oncology Nursing Care Scale
BMT: Bone marrow transplantation
CVR: Content validity ratio
CVI: Content validity index
SD: Standard deviation

## Author Contributions

Conceptualization: Raziyeh Ghafouri, Shiva Salar, and Malihe Nasiri. Data curating: Raziyeh Ghafouri, Shiva Salar, and Malihe Nasiri. Formal analysis: Raziyeh Ghafouri, Shiva Salar, and Malihe Nasiri. Investigation: Raziyeh Ghafouri, Shiva Salar, and Malihe Nasiri. Methodology: Raziyeh Ghafouri. Software: Raziyeh Ghafouri, Shiva Salar, and Malihe Nasiri. Supervision: Raziyeh Ghafouri. Validation: Raziyeh Ghafouri, Shiva Salar, and Malihe Nasiri. Writing–original draft: Raziyeh Ghafouri, Shiva Salar, and Malihe Nasiri. Writing–review and editing: Raziyeh Ghafouri, Shiva Salar, and Malihe Nasiri.

## Funding

This research was conducted without any external financial support.

## Disclosure

Relationship to a previous publication.

The present study was derived from a master’s thesis that generated two articles with distinct research objectives. The first article, which has been published, examined the psychometric properties of the Questionnaire on the Quality of Nursing Care in patients with hematological disorders. The present article compares the quality of nursing care between the primary nursing and case method models among patients with hematological malignancies. Both articles involved the same participants; however, they addressed different research questions. The published psychometric evaluation is cited in the Instruments section, and its findings are not reproduced as results in the present manuscript. All authors have read and approved the manuscript.

## Ethics Statement

This article is based on a Master’s thesis in Medical–Surgical Nursing. This study was reviewed and approved by the Research Ethics Committees of the School of Pharmacy and Nursing & Midwifery, Shahid Beheshti University of Medical Sciences (Ethics Code: IR.SBMU.PHARMACY.REC.1403.123) on 2024‐08‐05. The study was conducted in accordance with the principles outlined in the Declaration of Helsinki. All participants gave informed consent after being introduced to the study, receiving a clear explanation of its purpose, and being informed of their rights—including the right to withdraw at any time without affecting their treatment or care. Written informed consent was obtained from all patients before participation in the study. For patients who were unable to complete the consent process independently due to physical weakness, consent was obtained from both the patient and a family member, whenever feasible. Participation was voluntary, and confidentiality of all collected data was ensured. Participants were assured that their data would remain anonymous and confidential.

## Consent

Written informed consent for publication was obtained from the patients and their family members, where applicable.

## Conflicts of Interest

The authors declare no conflicts of interest.

## Supporting Information

Additional supporting information can be found online in the Supporting Information section.

## Supporting information


**Supporting Information 1** Supporting Information file1: Supporting tables for confirmatory factor analysis of the three‐factor model in hematology and bone marrow transplantation (BMT).

**Supporting Information 2** Supporting Information file 2: Excel file containing of research data.

## Data Availability

The datasets used and analyzed during the current study are available in Supporting file 2.
