# Supplementary material for: Comparison of Nursing Care Quality Between Team‐Based Primary Nursing and Case Method Approaches in Patients With Hematological Malignancies
Source: J Nurs Manag. 2026 Nov 9;2026:9634361. doi: 10.1155/jonm/9634361 (PMC13746098; doi:10.1155/jonm/9634361)
Supplement: Supplementary file 1 [file JONM-2026-9634361-s002.docx]

**Supplementary Material S1**:

**Supplementary Tables for Confirmatory Factor Analysis of the Three-Factor Model in Hematology and Bone Marrow Transplantation (BMT)**

**Table 1. Comparison of model fit indices between Hematology and BMT**

| **Fit index** | **Hematology** | **BMT** |
| --- | --- | --- |
| Chi-square (χ²) | 957.367 | 813.968 |
| Degrees of freedom (df) | 374 | 374 |
| χ²/df | 2.560 | 2.176 |
| Comparative Fit Index (CFI) | 0.782 | 0.793 |
| Tucker–Lewis Index (TLI) | 0.746 | 0.760 |
| Incremental Fit Index (IFI) | 0.787 | 0.799 |
| Normed Fit Index (NFI) | 0.692 | 0.683 |
| RMSEA | 0.135 | 0.117 |
| 90% CI for RMSEA | 0.125–0.146 | 0.106–0.128 |
| PCLOSE | 0.000 | 0.000 |
| Akaike Information Criterion (AIC) | 1137.367 | 993.968 |

Note. The three-factor model showed suboptimal fit in both groups. Although several fit indices were relatively better in BMT, these descriptive differences do not establish statistically significant differences between groups.

**Table 2. Standardized factor loadings by clinical group**

| **Factor** | **Item** | **Hematology** | **BMT** |
| --- | --- | --- | --- |
| Factor 1 | Q1 | 0.816 | 0.779 |
| Factor 1 | Q3 | 0.748 | 0.774 |
| Factor 1 | Q6 | 0.837 | 0.802 |
| Factor 1 | Q7 | 0.849 | 0.783 |
| Factor 1 | Q8 | 0.829 | 0.827 |
| Factor 1 | Q9 | 0.896 | 0.851 |
| Factor 1 | Q10 | 0.866 | 0.675 |
| Factor 1 | Q11 | 0.879 | 0.827 |
| Factor 1 | Q12 | 0.870 | 0.817 |
| Factor 1 | Q13 | 0.728 | 0.646 |
| Factor 1 | Q14 | 0.760 | 0.724 |
| Factor 1 | Q15 | 0.838 | 0.851 |
| Factor 1 | Q16 | 0.793 | 0.776 |
| Factor 2 | Q17 | 0.902 | 0.768 |
| Factor 2 | Q18 | 0.873 | 0.840 |
| Factor 2 | Q19 | 0.880 | 0.871 |
| Factor 2 | Q21 | 0.744 | 0.694 |
| Factor 2 | Q22 | 0.765 | 0.670 |
| Factor 2 | Q23 | 0.769 | 0.601 |
| Factor 2 | Q24 | 0.607 | 0.538 |
| Factor 2 | Q25 | 0.782 | 0.667 |
| Factor 2 | Q26 | 0.679 | 0.608 |
| Factor 3 | Q2 | 0.868 | 0.740 |
| Factor 3 | Q4 | 0.850 | 0.741 |
